# Supplementary material for: Tubular cell polyploidy protects from lethal acute kidney injury but promotes consequent chronic kidney disease
Source: Nat Commun. 2022 Oct 4;13:5805. doi: 10.1038/s41467-022-33110-5 (PMC9532438; doi:10.1038/s41467-022-33110-5)
Supplement: Supplementary file 1 — Supplementary Information [file 41467_2022_33110_MOESM1_ESM.pdf]

Tubular cell polyploidy protects from lethal Acute Kidney Injury but promotes consequent Chronic Kidney Disease

De Chiara L. et al.

Supplementary Information

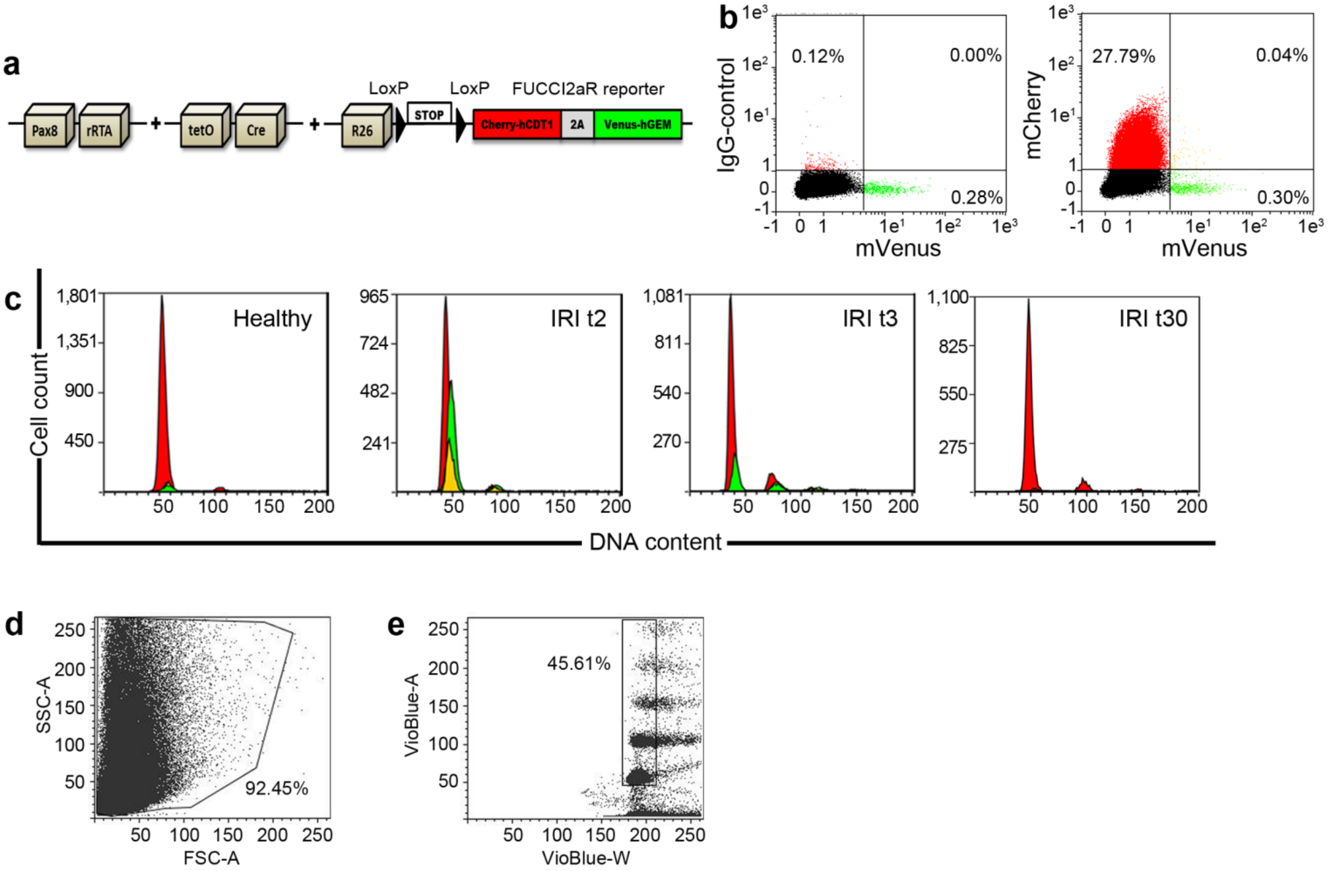

Supplementary Fig. 1. PAX8/FUCCI2aR mouse model and gating strategy. (a) The inducible Pax8.rtTA;TetO.Cre;R26.FUCCI2aR (Pax8/FUCCI2aR) mouse is produced by crossing the Pax8.rtTA transgenic mice with TetO.Cre and Rosa26-FUCCI2aR transgenic mice. Recombination is triggered upon doxycycline administration. (b) FACS analysis of healthy Pax8/FUCCI2aR mice (n=6). (c) Cell cycle distribution of mCherry$^+$, mVenus$^+$, and mCherry$^+$mVenus$^+$ TC in healthy and at day 2 (t2), 3 (t3) and 30 (t30) after ischemic reperfusion injury (IRI) in Pax8/FUCCI2aR mice. (d, e) Gating strategy to avoid counting cell doublets as a potential source of artifacts of freshly isolated total renal cells. A representative experiment out of 6 is shown.

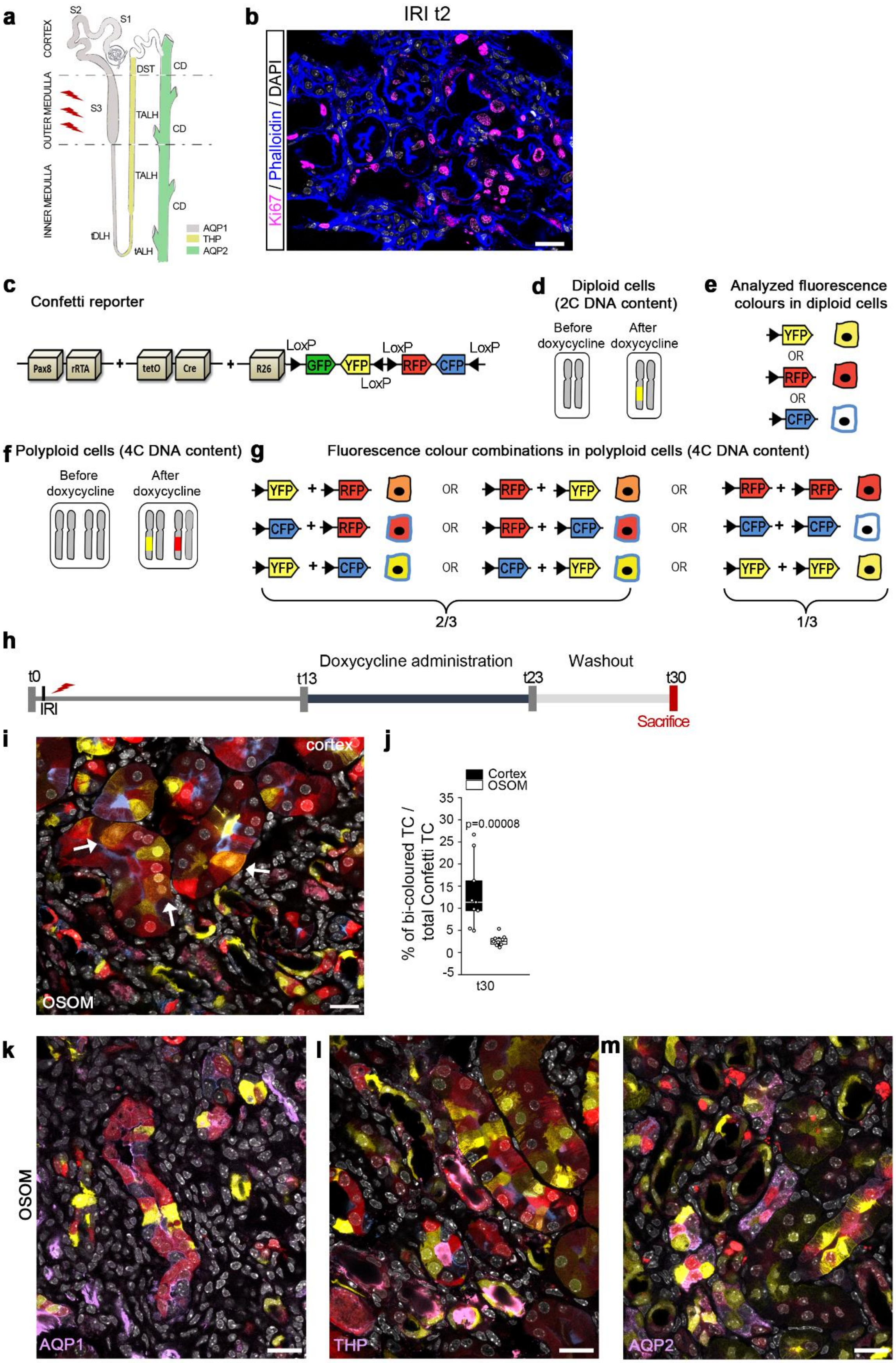
a
S2
S1
CORTEX
OUTER MEDULLA
INNER MEDULLA
DST
CD
S3
TALH
tDLH
tALH
AQP1
THP
AQP2
b
IRI t2
Ki67 / Phalloidin / DAPI
c
Confetti reporter
Pax8
rRTA
tetO
Cre
R26
LoxP
GFP
YFP
RFP
CFP
d
Diploid cells
(2C DNA content)
Before doxycycline
After doxycycline
e
Analyzed fluorescence colours in diploid cells
OR
f
Polyploid cells (4C DNA content)
g
Fluorescence colour combinations in polyploid cells (4C DNA content)
2/3
1/3
h
t0
IRI
t13
Doxycycline administration
t23
Washout
t30
Sacrifice
i
cortex
OSOM
j
Cortex
OSOM
p=0.00008
% of bi-coloured TC / total Confetti TC
t30
k
l
m
AQP1
THP
AQP2

Supplementary Fig. 2. Polyploid tubular cells (TC) can be identified by the Confetti reporter. (a) Schematic localization of injury in the S3 segment of the nephron. (b) Representative picture showing Ki67^{+} Phalloidin^{+} TC 2 days (t2) after ischemic reperfusion injury (IRI). DAPI counterstains nuclei. Bar 25µm. A representative experiment out of 3 is shown. (c) The inducible heterozygous Pax8.rtTA;TetO.Cre;R26.Confetti (Pax8/Confetti) mouse is produced by crossing the Pax8.rtTA transgenic mice with TetO.Cre and Rosa26-Confetti transgenic mice. Recombination is triggered upon doxycycline administration. (d) Schematic representation of the Confetti reporter-based model in diploid TC before and after doxycycline administration in heterozygous Pax8/Confetti mice. (e) Analyzed fluorescence colours (yellow, YFP, red, RFP and cyan, CFP) in diploid TC. (f) Schematic representation of the Confetti reporter-based model in polyploid TC before and after doxycycline administration in heterozygous Pax8/Confetti mice. (g) Possible fluorescence colour combinations in polyploid TC with 4C DNA content, when the three fluorescence colours (YFP, RFP, CFP) are evaluated. Numbers indicate the expected probability of bi-coloured TC and mono-coloured TC. Fluorescence colour combinations that generate bi-coloured polyploid TC were evaluated. (h) Schematic representation of doxycycline schedule in Pax8/Confetti mice after IRI. Sham-control mice underwent the same doxycycline schedule without IRI. (i) Representative picture of the cortex and the OSOM in a heterozygous Pax8/Confetti mouse showing distribution of polyploid TC. Arrows indicate bi-coloured polyploid TC. DAPI counterstains nuclei. Bar 20µm. (j) Percentage of bi-coloured TC on total Confetti TC localized in the cortex or in the OSOM of heterozygous Pax8/Confetti mice after IRI (n=9). (k) AQP1, (l) THP and (m) AQP2 staining in the OSOM of heterozygous Pax8/Confetti mice 30 days after IRI. DAPI counterstains nuclei. Bar 20 µm. S1: S1 segment of proximal tubule, S2: S2 segment of proximal tubule, S3: S3 segment of proximal tubule, tDLH: thin descending limb of loop of Henle, tALH: thin ascending limb of loop of Henle, TALH: thick ascending limb of loop of Henle, DST: distal straight tubule, CD: collecting duct. OSOM: outer stripe of outer medulla; AQP1: Aquaporin-1; THP: Tamm-Horsfall

protein; AQP2: Aquaporin-2. Statistical significance was calculated by two-sided Mann-Whitney test; number on graph represent exact p value. Box-and-whisker plots: line = median, box =25-75%, whiskers = outlier (coef. 1.5).

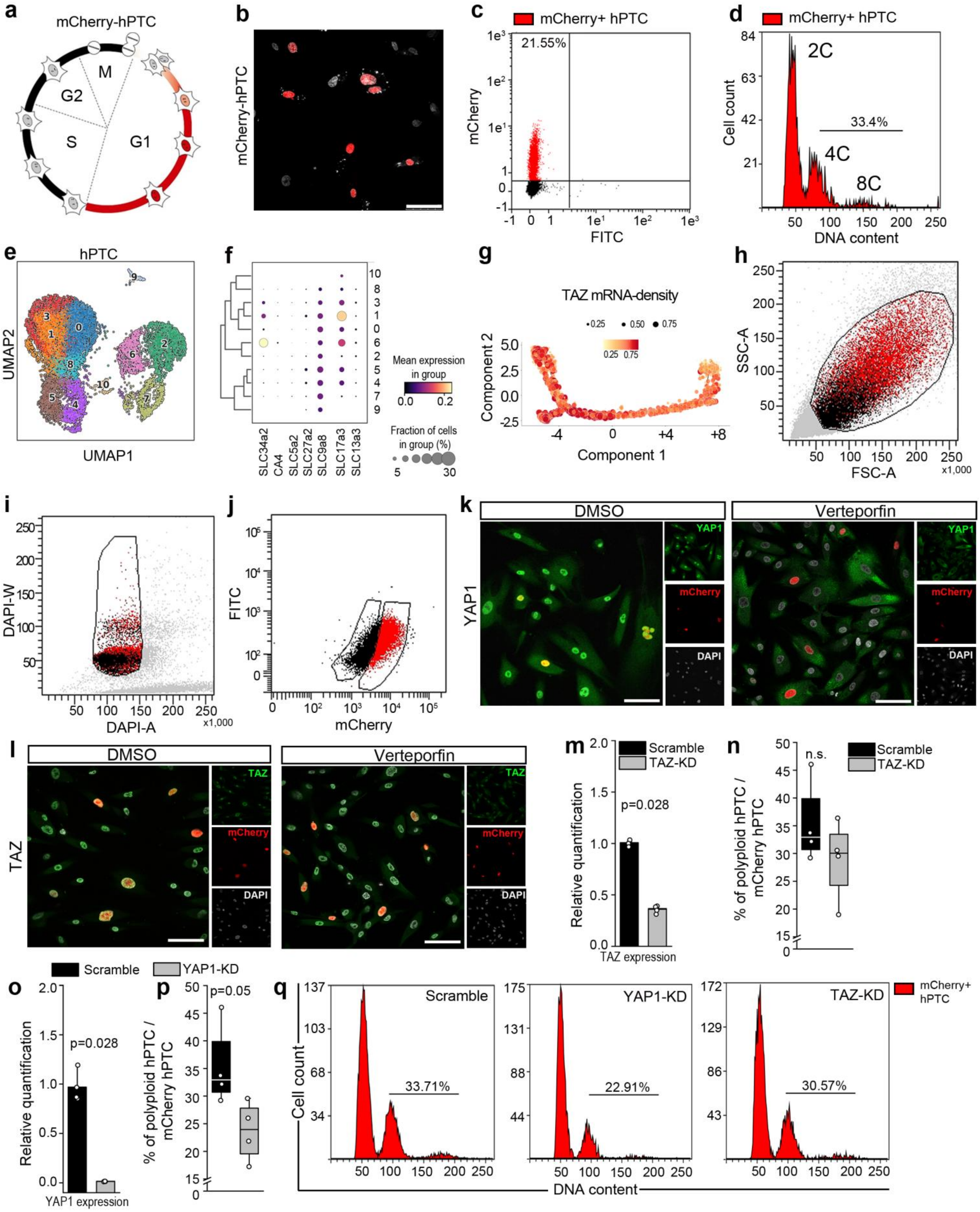
a
mCherry-hPTC
M
G2
S
G1
b
mCherry-hPTC
c
mCherry+ hPTC
21.55%
mCherry
FITC
d
mCherry+ hPTC
2C
4C
8C
33.4%
Cell count
DNA content
e
hPTC
UMAP2
UMAP1
f
SLC34a2
CA4
SLC5a2
SLC27a2
SLC9a8
SLC17a3
SLC13a3
Mean expression in group
Fraction of cells in group (%)
g
TAZ mRNA-density
Component 2
Component 1
h
SSC-A
FSC-A
x1,000
i
DAPI-W
DAPI-A
x1,000
j
FITC
mCherry
k
DMSO
Verteporfin
YAP1
mCherry
DAPI
l
DMSO
Verteporfin
TAZ
mCherry
DAPI
m
Scramble
TAZ-KD
Relative quantification
p=0.028
TAZ expression
n
n.s.
Scramble
TAZ-KD
% of polyploid hPTC / mCherry hPTC
o
Scramble
YAP1-KD
Relative quantification
p=0.028
YAP1 expression
p
p=0.05
% of polyploid hPTC / mCherry hPTC
q
Scramble
33.71%
YAP1-KD
22.91%
TAZ-KD
30.57%
mCherry+ hPTC
Cell count
DNA content

Supplementary Fig. 3. Human proximal tubular cell (hPTC) polyploidization is controlled by YAP1. (a) Scheme of the pRetroX-G1-Red (mCherry-G1) retrovirus. G1, gap 1 phase; S, synthesis; G2, gap 2 phase; M, mitosis. (b) Representative picture of mCherry-hPTC. DAPI counterstains nuclei. Bar 75µm. (c) FACS analysis of mCherry-hPTC. An experiment out of 6 is shown. (d) Cell cycle distribution of mCherry-hPTC showing DNA content. A representative experiment out of 6 is shown. (e) UMAP showing hPTC clusters. (f) Dot plot of solute carrier family (Slc) gene expression. Based on the Slc expression, clusters 1 and 6 were excluded from the trajectory analysis as they belong to other portions of the nephron. (g) Monocle2 trajectory of hPTC coloured by pseudotime showing TAZ mRNA density. (h-j) FACS analysis of mCherry-hPTC showing the gating strategy for sorting. A representative experiment out of 4 is shown. (k) YAP1 immunofluorescence in mCherry-hPTC treated with DMSO or verteporfin. DAPI counterstains nuclei. Bars 75µm. A representative experiment out of 6 is shown. (l) TAZ immunofluorescence in mCherry-hPTC treated with DMSO or verteporfin. DAPI counterstains nuclei. Bars 75µm. A representative experiment out of 4 is shown. (m) Knock-down (KD) efficiency of TAZ GapmeRs (n=4). (n) Percentage of polyploid mCherry-hPTC in TAZ-KD or scramble-treated culture (n=4). (o) KD efficiency of YAP1 GapmeRs (n=4). (p) Percentage of polyploid mCherry-hPTC in YAP1-KD or scramble-treated culture (n=4). (q) Cell cycle distribution of mCherry-hPTC transfected with scramble, YAP1 or TAZ GapmeRs. A representative experiment out of 4 is shown. Statistical significance was calculated by two-sided Mann-Whitney test; numbers on graphs represent exact p values. Box-and-whisker plots: line = median, box =25-75%, whiskers = outlier (coef. 1.5). Bar plots: line = mean, whiskers = outlier (coef. 1.5).

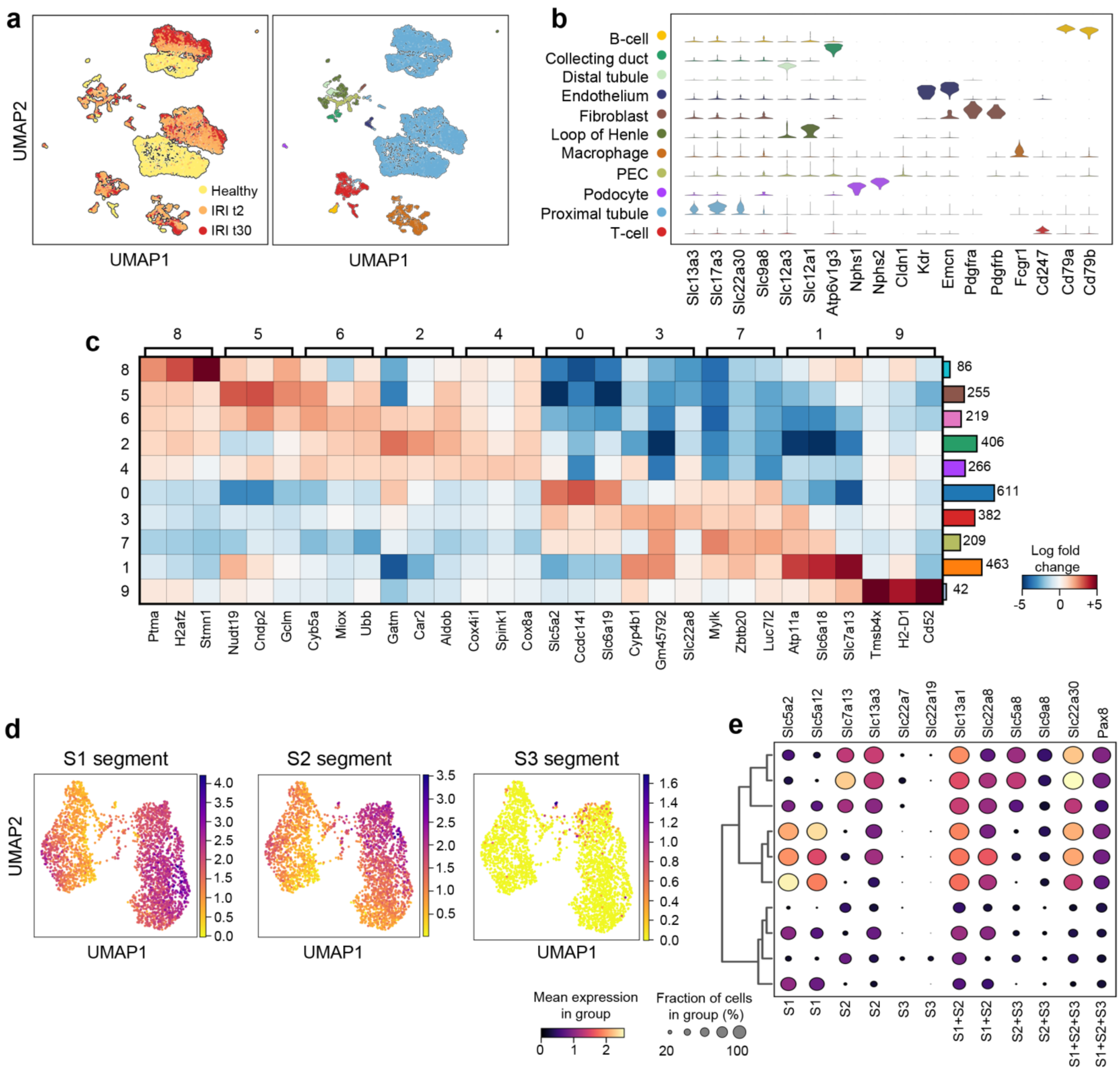

Supplementary Fig. 4. Single cell RNA-sequencing on mouse kidney after ischemic injury. (a) UMAP of sample distribution and cell population retrieved in healthy kidney (n=1) and at day 2 (t2, n=2) and 30 (t30, n=3) after ischemic reperfusion injury (IRI). (b) Violin plot displaying gene expression patterns of cluster-enriched markers. (c) Matrix plot showing the top three cluster marker genes. Numbers indicate the number of cells composing each cluster. (d) UMAP of gene distribution for S1, S2 and S3 segments. (e) Dot plot of solute carrier family (Slc) gene expression identifying S1, S2 and S3 segments.

S1: S1 segment of proximal tubule, S2: S2 segment of proximal tubule, S3: S3 segment of proximal tubule.

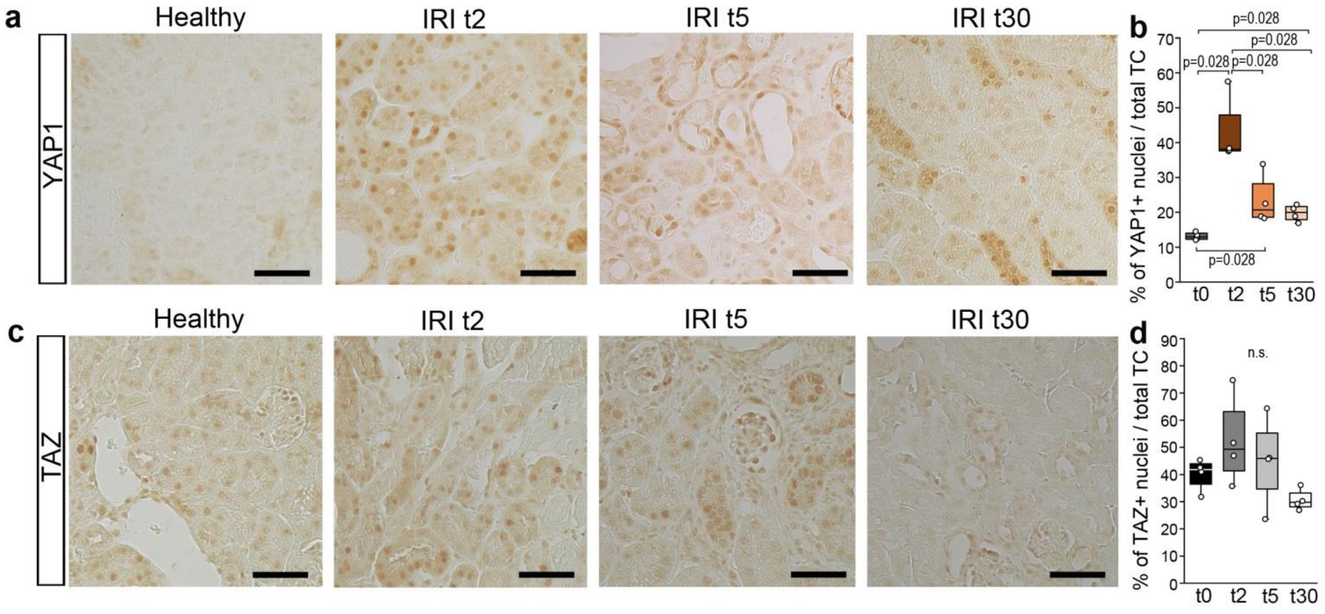

Supplementary Fig. 5. YAP1 but not TAZ is upregulated following ischemic injury. (a) Representative pictures of YAP1 expression in healthy kidney and at day 2, 5 and 30 after IRI. Bars 50µm. (b) Percentage of YAP1+ TC nuclei on total TC in healthy kidney (t0) and at day 2, 5 and 30 after IRI (n=4). (c) Representative pictures of TAZ expression in healthy kidney and at day 2, 5 and 30 after IRI. Bars 50µm. (d) Percentage of TAZ+ TC nuclei on total TC in healthy kidney (t0) and at day 2, 5 and 30 after IRI (n=4). IRI: ischemia reperfusion injury. t2: day2 after IRI; t5: day 5 after IRI; t30: day 30 after IRI. Statistical significance was calculated by two-sided Mann-Whitney test; numbers on graph represent exact p values. Box-and-whisker plots: line = median, box =25-75%, whiskers = outlier (coef. 1.5).

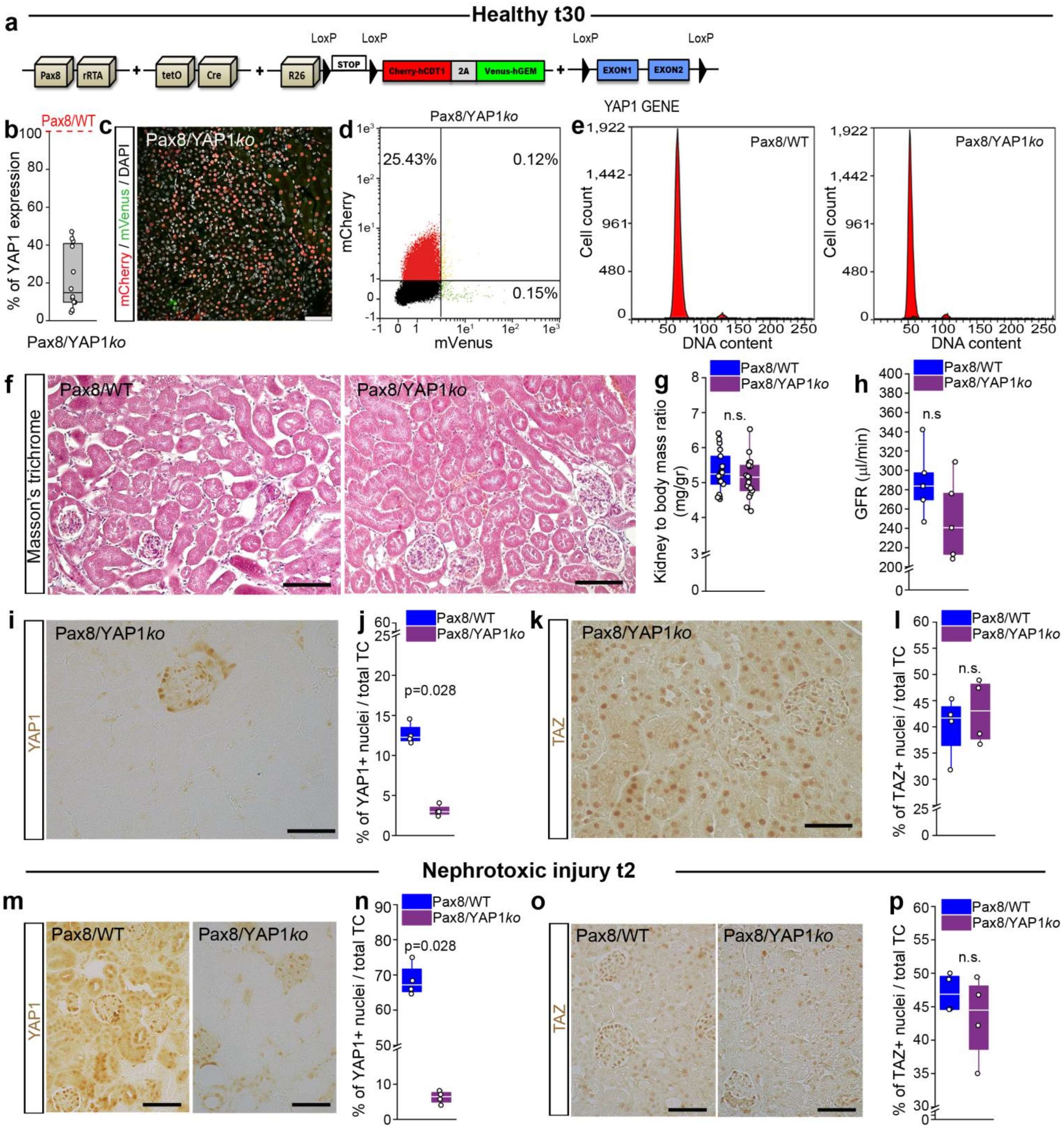

Supplementary Fig. 6. YAP1 but not TAZ is differentially expressed in Pax8/YAP1*ko*. (a) The inducible Pax8.rtTA;TetO.Cre;R26.FUCCI2aR;YAP1knock-out (Pax8/YAP1*ko*) mouse is produced by crossing the Pax8.rtTA transgenic mice with TetO.Cre and Rosa26-FUCCI2aR transgenic mice with

YAP1$^{fl/fl}$. Recombination is triggered upon doxycycline administration. (b) YAP1 mRNA expression by qRT-PCR analysis. Percentage of YAP1 expression is normalized on YAP1 expression in healthy kidneys (n=10 replicates are shown). (c) Representative picture of healthy Pax8/YAP1*ko*, showing colours of the reporter. DAPI counterstains nuclei. Bar 50µm. A representative experiment out of 4 is shown. (d) FACS analysis of Pax8/YAP1*ko* mice (n=6). (e) Cell cycle distribution of mCherry$^+$, mVenus$^+$, and mCherry$^+$mVenus$^+$ tubular cells (TC) in healthy Pax8/WT mice and in Pax8/YAP1*ko* mice (n=6). (f) Representative pictures of Masson's trichrome of healthy Pax8/WT and Pax8/YAP1*ko* mice (n=5). Bars 100µm. (g) Kidney weight on total body weight in healthy Pax8/WT and in Pax8/YAP1*ko* mice (n=18). (h) Glomerular filtration rate (GFR) measurement at day 30 after doxycycline induction in healthy Pax8/WT and in Pax8/YAP1*ko* mice (n=5). (i) Representative picture of YAP1 expression in healthy Pax8/YAP1*ko* mice. Bar 50µm. (j) Percentage of YAP1+ TC nuclei on total TC in healthy Pax8/WT and in Pax8/YAP1*ko* mice (n=4). (k) Representative picture of TAZ expression in healthy Pax8/YAP1*ko* mice. Bar 50µm. (l) Percentage of TAZ+ TC nuclei on total TC in healthy Pax8/WT and in Pax8/YAP1*ko* mice (n=4). (m) Representative pictures of YAP1 expression in Pax8/WT and in Pax8/YAP1*ko* mice 2 days after nephrotoxic AKI. Bars 50µm. (n) Percentage of YAP1+ TC nuclei on total TC in healthy Pax8/WT and Pax8/YAP1*ko* mice 2 days after nephrotoxic AKI (n=4). (o) Representative pictures of TAZ expression in Pax8/WT and in Pax8/YAP1*ko* mice 2 days after nephrotoxic AKI. Bars 50µm. (p) Percentage of TAZ+ TC nuclei on total TC in healthy Pax8/WT and in Pax8/YAP1*ko* mice 2 days after nephrotoxic AKI (n=4). t30: 30 days after doxycycline induction, t2: 2 days after nephrotoxic injury. Statistical significance was calculated by two-sided Mann-Whitney test; numbers on graphs represent exact p values. Box-and-whisker plots: line = median, box =25-75%, whiskers = outlier (coef. 1.5).

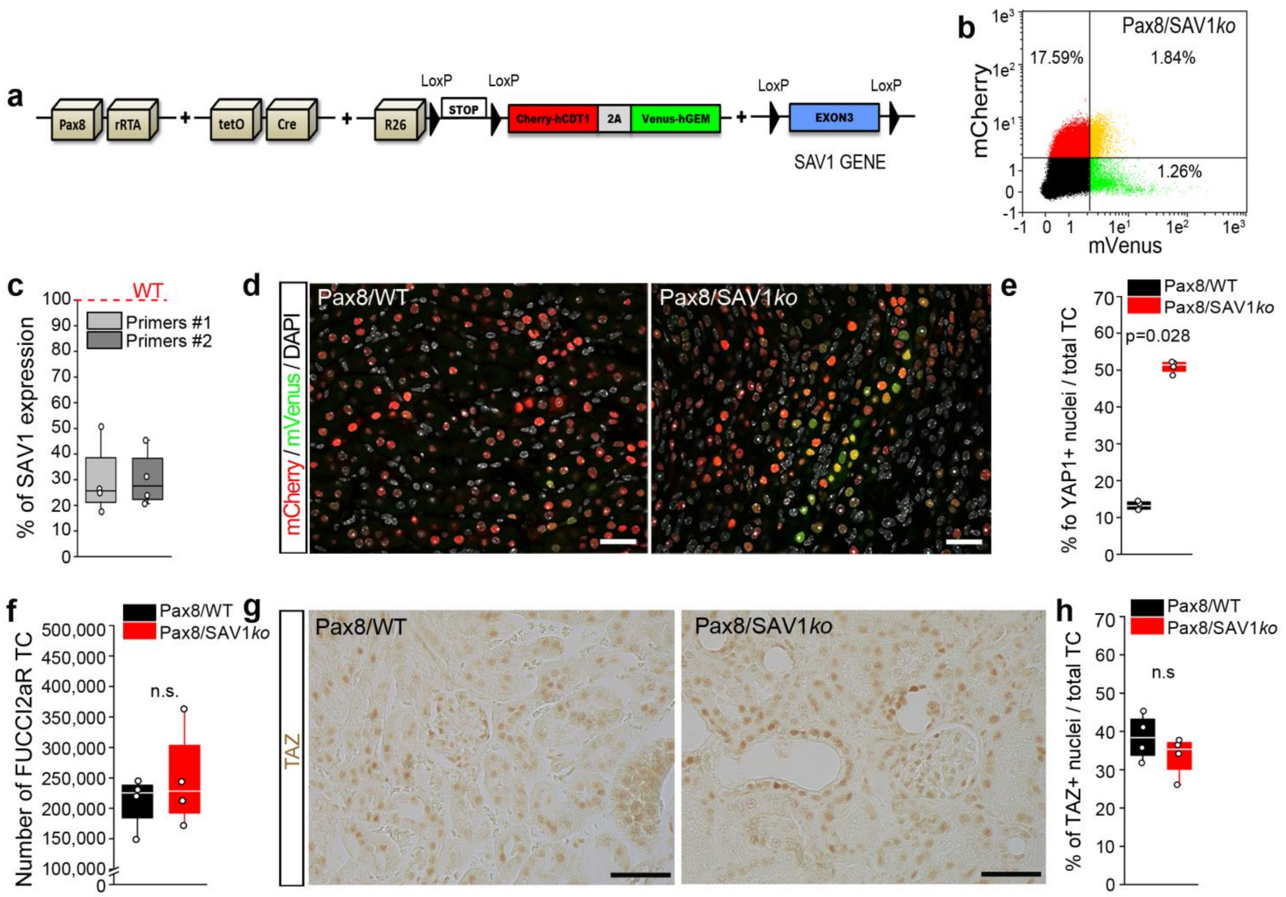

Supplementary Fig. 7. Generation of Pax8/FUCCI2aR/SAV1 knock-out mice (Pax8/SAV1*ko*). (a) The inducible Pax8.rtTA;TetO.Cre;R26.FUCCI2aR;SAV1knock-out (Pax8/SAV1*ko*) mouse is produced by crossing the Pax8.rtTA transgenic mice with TetO.Cre and Rosa26-FUCCI2aR transgenic mice with *SAV1*$^{fl/fl}$. (b) FACS analysis of healthy Pax8/SAV1*ko* mice (n=8). (c) SAV1 mRNA expression in kidney tissue of Pax8/SAV1*ko* analysed using two different sets of primers by qRT-PCR. SAV1 expression is normalized on SAV1 expression of healthy kidneys (n=4 replicates are shown). (d) Representative pictures of healthy Pax8/WT and Pax8/SAV1*ko* mice, showing colours of the reporter. DAPI counterstains nuclei. Bars 25µm. A representative experiment out of 4 is shown. (e) Percentage of YAP1+ tubular cell (TC) nuclei on total TC in healthy Pax8/WT and in Pax8/SAV1*ko* mice (n=4). (f) Total number of FUCCI2aR TC in healthy Pax8/WT and in Pax8/SAV1*ko* mice at day 30 after doxycycline induction (n=4). (g) Representative pictures of TAZ expression in healthy Pax8/WT and in Pax8/SAV1*ko*

mice. Bars 100µm. (h) Percentage of TAZ+ TC nuclei on total TC in healthy Pax8/WT and Pax8/SAV1*ko* mice (n=4). Statistical significance was calculated by two-sided Mann-Whitney test; number on graph represent exact p value. Box-and-whisker plots: line = median, box =25-75%, whisker = outlier (coef. 1.5).

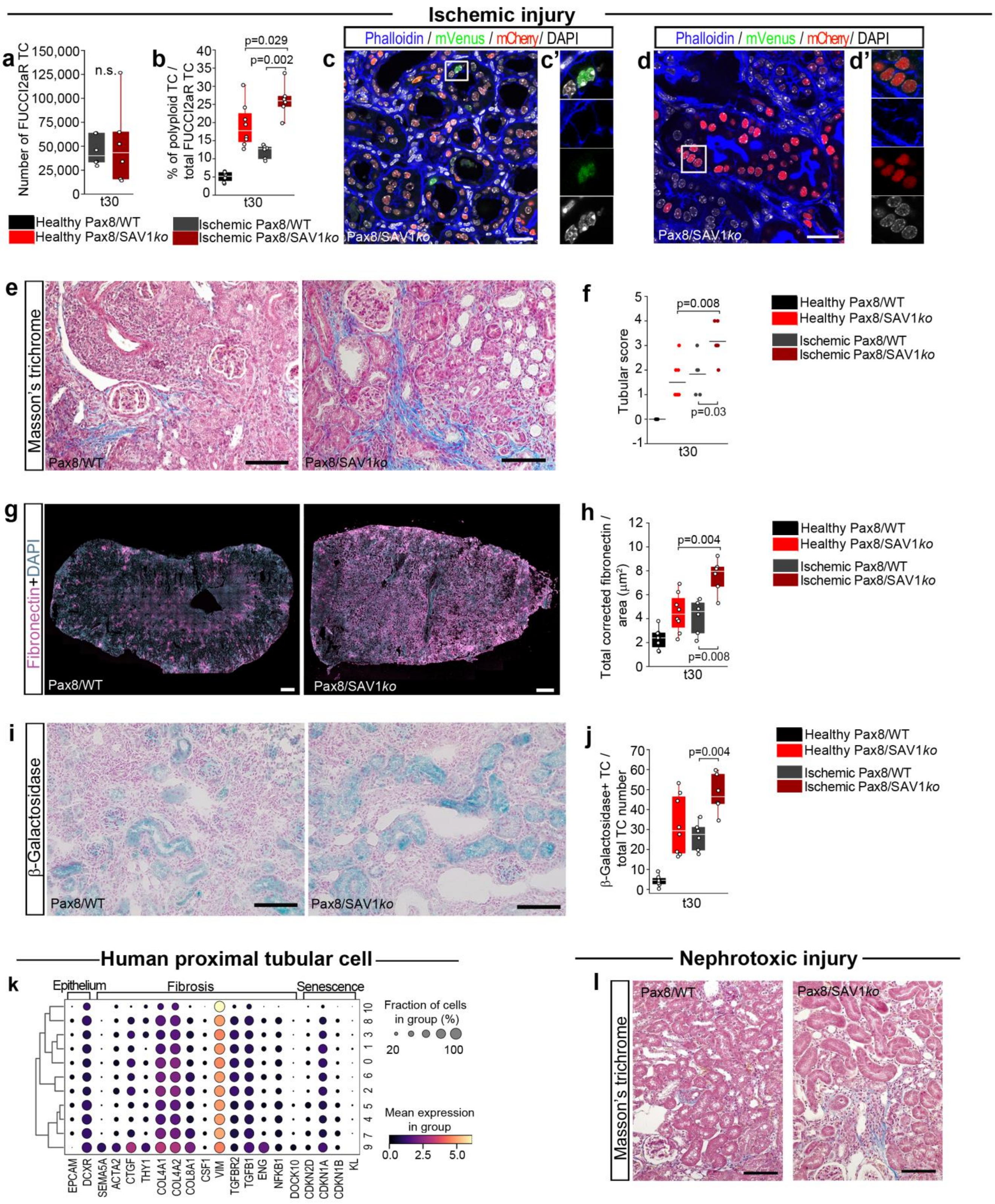
Ischemic injury
a
Number of FUCCI2aR TC
n.s.
t30
b
% of polyploid TC / total FUCCI2aR TC
p=0.029
p=0.002
t30
Healthy Pax8/WT
Healthy Pax8/SAV1ko
Ischemic Pax8/WT
Ischemic Pax8/SAV1ko
c
c'
Phalloidin / mVenus / mCherry/ DAPI
Pax8/SAV1ko
d
d'
Phalloidin / mVenus / mCherry/ DAPI
Pax8/SAV1ko
e
Masson's trichrome
Pax8/WT
Pax8/SAV1ko
f
Tubular score
p=0.008
p=0.03
t30
g
Fibronectin+DAPI
Pax8/WT
Pax8/SAV1ko
h
Total corrected fibronectin / area (μm²)
p=0.004
p=0.008
t30
i
β-Galactosidase
Pax8/WT
Pax8/SAV1ko
j
β-Galactosidase+ TC / total TC number
p=0.004
t30
Human proximal tubular cell
k
Epithelium
Fibrosis
Senescence
EPCAM DCXR SEMA5A ACTA2 CTGF THY1 COL4A1 COL4A2 COL8A1 CSF1 VIM TGFBR2 TGFB1 ENG NFKB1 DOCK10 CDKN2D CDKN1A CDKN1B KL
Fraction of cells in group (%)
20
100
Mean expression in group
0.0 2.5 5.0
Nephrotoxic injury
l
Masson's trichrome
Pax8/WT
Pax8/SAV1ko

Supplementary Fig. 8. Acute kidney injury exacerbates polyploidy, fibrosis and senescence in Pax8/SAV1*ko* mice. (a) Total number of FUCCI2aR tubular cells (TC) in Pax8/WT and in Pax8/SAV1*ko* mice at day 30 after ischemic reperfusion injury (IRI) (n=6). (b) Percentage of polyploid TC on the total number of FUCCI2aR TC in healthy Pax8/WT mice and in Pax8/SAV1*ko* mice (n=8) and at day 30 after IRI (n=6). (c, d) Representative pictures showing TC undergoing endomitosis (two mVenus^{+} or mCherry^{+} nuclei in the same cell) in Pax8/SAV1*ko* mice at day 30 after IRI. Phalloidin stains TC and DAPI counterstains nuclei. Bars 25µm. A representative experiment out of 6 is shown. (c', d') Higher magnifications of the inlets shown in (c) and (d) respectively. (e) Representative pictures of Masson's trichrome staining in Pax8/WT and in Pax8/ SAV1*ko* mice at day 30 after IRI (n=6). Bars 100µm. (f) Tubular score evaluated on Masson's trichrome staining in healthy Pax8/WT mice (n=8) and in Pax8/SAV1*ko* mice and at day 30 after IRI (n=6). (g) Sequential scanning of whole kidney sections stained for fibronectin in Pax8/WT and in Pax8/SAV1*ko* mice at day 30 after IRI (n=6). DAPI counterstains nuclei. Bars 500µm. (h) Quantification of fibronectin deposition by digital morphometry in healthy Pax8/WT mice and in Pax8/SAV1*ko* mice (n=8) and at day 30 after IRI (n=6). (i) Senescence-associated β-galactosidase assay in Pax8/WT and Pax8/SAV1*ko* snap-frozen sections at day 30 after IRI (n=6). Bars 100µm. (j) Percentage of β-galactosidase^{+} TC on the total TC number in healthy Pax8/WT and in Pax8/SAV1*ko* mice (n=8) and at day 30 after IRI (n=6). (k) Dot plot showing epithelial, fibrotic and senescence gene expression in human proximal tubular cell clusters. (l) Representative pictures of Masson's trichrome staining in Pax8/WT and Pax8/SAV1*ko* mice at day 30 after nephrotoxic injury (n=8). Bars 100µm. Statistical significance was calculated by two-sided Mann-Whitney test; numbers on graphs represent exact p values. Box-and-whisker plots: line = median, box =25-75%, whiskers = outlier (coef. 1.5).

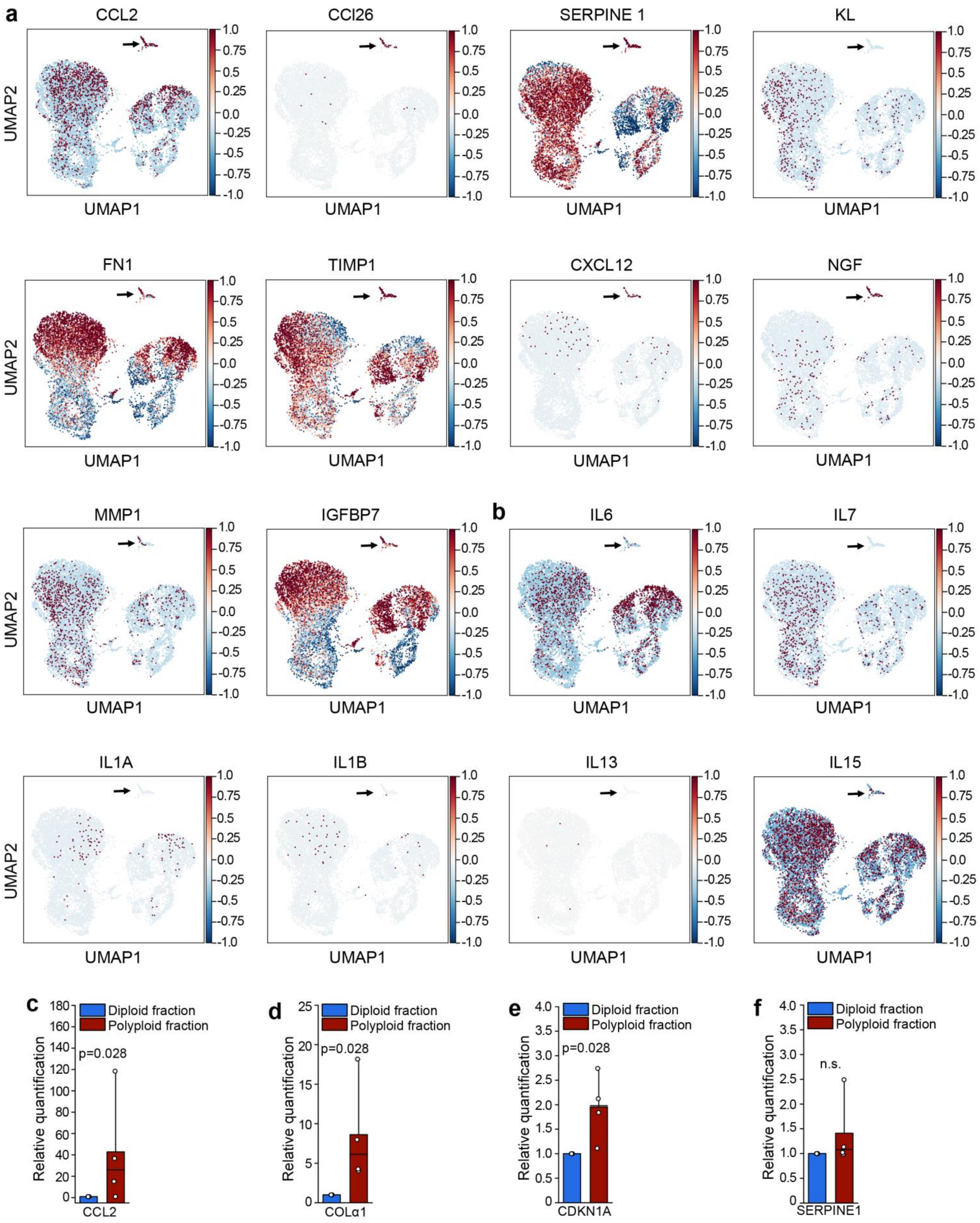
a
CCL2
CCl26
SERPINE 1
KL
FN1
TIMP1
CXCL12
NGF
MMP1
IGFBP7
b
IL6
IL7
IL1A
IL1B
IL13
IL15
UMAP1
UMAP2
1.0
0.75
0.5
0.25
0.0
-0.25
-0.5
-0.75
-1.0
c
d
e
f
Diploid fraction
Polyploid fraction
Relative quantification
p=0.028
n.s.
CCL2
COLα1
CDKN1A
SERPINE1

Supplementary Fig. 9. Senescence-associated secretory phenotype (SASP) of polyploid tubular cells (TC). (a) UMAP of gene distribution associated with SASP in human proximal tubular cells (hPTC). Arrows indicate polyploid hPTC cluster 9. (b) UMAP of interleukin gene distribution showing that polyploid TC do not express interleukins. (c-f) SASP-associated gene expression of sorted polyploid mCherry-hPTC and diploid mCherry-hPTC (n=4). Statistical significance was calculated by two-sided Mann-Whitney test; numbers on graphs represent exact p values. Bar plots: line = mean, whisker = outlier (coef. 1.5)

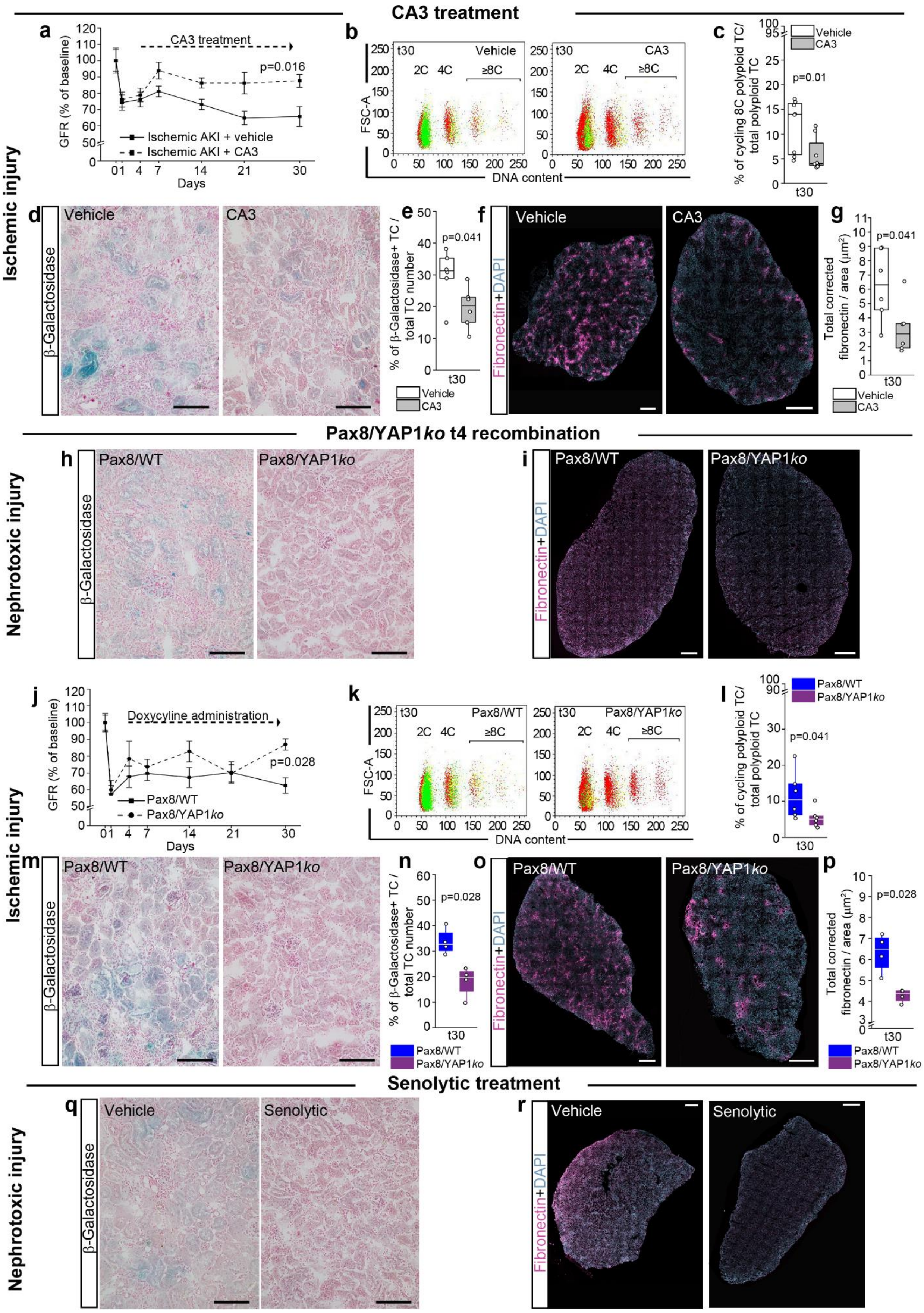
CA3 treatment
Ischemic injury
CA3 treatment
p=0.016
Ischemic AKI + vehicle
Ischemic AKI + CA3
GFR (% of baseline)
Days
DNA content
FSC-A
2C
4C
≥8C
Vehicle
CA3
p=0.01
% of cycling 8C polyploid TC/ total polyploid TC
β-Galactosidase
% of β-Galactosidase+ TC / total TC number
p=0.041
Fibronectin+DAPI
Total corrected fibronectin / area (μm²)
Pax8/YAP1ko t4 recombination
Nephrotoxic injury
Pax8/WT
Pax8/YAP1ko
Ischemic injury
Doxycyline administration
p=0.028
% of cycling polyploid TC/ total polyploid TC
Senolytic treatment
Senolytic

Supplementary Fig. 10. Time-dependent inhibition of tubular cell (TC) polyploidization attenuates AKI-CKD transition. (a) Glomerular filtration rate **(**GFR) measurement in WT mice after ischemic injury (n=6). (b) FACS plots of TC in Pax8/FUCCI2aR mice after ischemic injury, showing diploid (2C), tetraploid (4C) and octaploid or greater (≥8C) TC (n=8). Colours match the FUCCI2aR reporter. (c) Percentage of cycling polyploid (≥8C) TC on total polyploid TC in Pax8/FUCCI2aR mice after ischemic injury (n=8). (d) Senescence-associated β-galactosidase assay in WT mice snap-frozen sections at day 30 after ischemic injury (n=6). Bars 100µm. (e) Percentage of β-galactosidase+ TC in WT mice after ischemic injury (n=6). (f) Sequential scanning of kidney sections stained for fibronectin in WT mice at day 30 after ischemic injury (n=6). Bars 500µm. DAPI counterstains nuclei. (g) Quantification of fibronectin deposition by digital morphometry in WT mice after ischemic injury (n=6). (h) Senescence-associated β-galactosidase assay in Pax8/WT and in Pax8/YAP1*ko* snap-frozen sections 30 days after nephrotoxic injury (n=8). Bars 100µm. (i) Sequential scanning of kidney sections stained for fibronectin in Pax8/WT and in Pax8/YAP1*ko* at day 30 after nephrotoxic injury (n=8). Bars 500µm. DAPI counterstains nuclei. (j) GFR measurement after ischemic injury (n=4). (k) FACS plots of TC in Pax8/WT and in Pax8/YAP1*ko* mice after ischemic injury, showing diploid (2C), tetraploid (4C) and octaploid or greater (≥8C) TC (n=6). Colours match the FUCCI2aR reporter. (l) Percentage of cycling polyploid (≥8C) TC on total polyploid TC in Pax8/WT and in Pax8/YAP1*ko* mice after ischemic injury (n=6). (m) Senescence-associated β-galactosidase assay in Pax8/WT and Pax8/YAP1*ko* snap-frozen sections 30 days after ischemic injury (n=4). Bars 100µm. (n) Percentage of β-galactosidase+ TC after ischemic injury (n=4). (o) Sequential scanning of kidney sections stained for fibronectin in Pax8/WT and in Pax8/YAP1*ko* at day 30 after ischemic injury (n=4). Bars 500µm. DAPI counterstains nuclei. (p) Quantification of fibronectin deposition by digital morphometry after ischemic injury (n=4). (q) Senescence-associated β-galactosidase assay 30 days after nephrotoxic injury (n=7). Bars 100µm. (r) Sequential scanning of kidney sections stained for fibronectin at day 30 after nephrotoxic injury (n=7).

Bars 500µm. DAPI counterstains nuclei. t4 recombination: doxycycline was given 4 days after nephrotoxic injury. Statistical significance was calculated by two-sided Mann-Whitney test; numbers on graphs represent exact p values. Two-way ANOVA test of significance followed by Bonferroni post-test was employed for graph a. Data are expressed as mean±SEM in graph a and j. Box-and-whisker plots: line = median, box =25-75%, whiskers = outlier (coef. 1.5).

Supplementary Tables

	0	1	2	3	4	5	6	7	8	9	10
1	IL32	VCAM1	PDLIM4	IFITM3	CDC20	ATAD2	MAL	HMGB1	TUBA1B	COL3A1	RPL19
2	NRCAM	SPP1	LIMA1	ITM2B	CCNB1	MCM3	IGFBP7	STMN1	ENO1	SRGN	RPL14
3	ITGAV	DAB2	MMP2	NNMT	CENPF	GINS2	TFAP2B	SMC4	IGFBP4	COL1A2	RPL6
4	ALCAM	ADIRF	HOXB9	FN1	PTTG1	CLSPN	PDLIM4	PCLAF	LMNA	COL1A1	RPS24
5	MFGE8	PDZK1IP1	KRT19	FTL	CDKN3	MCM7	CD24	PTMA	HMGN2	NR2F1	RACK1
6	CDH6	CRYAB	L1CAM	CST3	DLGAP5	FEN1	SAT1	TYMS	NPM1	COL5A2	RPL7A
7	COL18A1	CDH6	HSPG2	NEAT1	TPX2	PCNA	IRX2	DEK	HNRNPC	SPARC	EEF1B2
8	PLAU	MT1E	GSTP1	LTBP3	CKS2	TYMS	TPM2	CENPF	KRT18	BGN	RPS23
9	TPT1	HIF1A	CD81	HIF1A	H2AFZ	MCM4	TFCP2L1	MKI67	HSPD1	IGFBP6	RPS3A
10	PADI2	SERPINE2	TPM2	AKR1C3	BIRC5	MCM5	IGFBP2	NUCKS1	YBX1	TAGLN	RPL5
11	AL355916.1	TNFSF10	LOXL2	IGFBP7	MKI67	NASP	SERPINE2	TK1	PPP1R14B	TPM2	S100A10
12	TPM1	KCNJ15	RHOBTB3	MALAT1	UBE2S	HELLS	FKBP10	TPX2	HNRNPM	RCN3	RPL9
13	OPTN	TINAGL1	FCGRT	HTRA1	NUSAP1	DNAJC9	CD59	NUSAP1	EIF5A	COL5A1	RPL13
14	PXDN	SERPINA1	AMIGO2	SPP1	HMMR	TK1	GPNMB	MYBL2	PRDX1	SERPINH1	RPLP1
15	FN1	PTER	S100A11	SERPINA1	TUBB4B	PCLAF	LGALS3	ANLN	HMGB1	FSTL1	RPL15
16	ZBTB20	ABCC3	HOXB3	PDZK1IP1	CCNB2	HIST1H4C	HIPK2	CKS1B	ACTB	CKAP4	RPS4X
17	MACF1	MPC2	COL1A1	CLU	HMGB2	MYBL2	FHL2	TOP2A	XRCC6	COL12A1	RPS27A
18	SVIL	SLC9A3R1	GAS6	CTSB	CEP55	DEK	DCN	H2AFZ	HNRNPA1	LMO7	VIM
19	EEF1A1	TRIM38	GSN	SLC6A6	TUBA1C	H2AFZ	GYPC	HSP90AA1	PTMA	TIMP1	RPL10
20	RAB3B	PLA2G16	FHL2	GPX3	ANLN	DNMT1	SIM2	TUBB	RPL22L1	DDR2	RPL11
21	RRAS2	ANPEP	COL7A1	HLA-A	KPNA2	BRCA1	BRI3	ASPM	PLK2	COL6A2	RPS7
22	S100A6	CTSB	PTBP3	IFITM2	HMGB1	RRM2	EVA1B	TUBA1B	HNRNPA2B1	LRRC17	RPS13
23	ADGRG1	SIGIRR	ADAMTS5	SLC7A7	SMC4	GMNN	TCIM	NASP	PRELID1	CCDC80	RPL27
24	PRDX5	ADGRG1	NDN	RPL3	HMGN2	UBE2T	CTSD	CENPW	PSMB1	COL6A3	RPL35A
25	AMIGO2	RNASET2	CSRP1	MT1X	MAD2L1	ORC6	FTH1	CDKN3	HNRNPD	CALU	FAU
26	ATP5F1E	C19orf33	THBS1	NUPR1	DTYMK	HSPD1	PTH1R	DTYMK	HNRNPH3	ENG	RPL35
27	MYL9	AIF1L	SOX4	CGNL1	PTGES3	PPM1G	HOXB3	TMPO	PRMT1	MEG3	RPL13A
28	AP1M2	ITGA3	TACSTD2	VCAM1	CCNA2	DHFR	GSN	RANBP1	OAZ1	POSTN	RPS18
29	JUP	MGST1	FBLIM1	ABCC3	BUB1	USP1	SFRP1	HMGB2	HSPE1	MARCKS	RPL23A
30	WWTR1	PLOD2	BICD1	CRYAB	PBK	TMPO	CD81	BIRC5	PPIA	CAVIN1	RPL10A
31	LTBP2	MT1X	SRP14	TIMP1	CKS1B	HNRNPAB	KCNJ16	RRM2	TUBB4B	MYL9	RPL32
32	VAT1	LINC01781	TAGLN	TKT	TOP2A	FAM111B	FBXO32	PTTG1	HSPA5	ISLR	NACA
33	SPINT1	SCEL	LUM	UGT2B7	ASPM	DUT	CD9	HMGN2	C1QBP	COL6A1	RPL12
34	DCBLD2	THBS2	TMSB4X	MMP14	JPT1	CDT1	GMDS	KPNA2	FABP5	MEST	RPL21
35	AHNAK2	CHCHD10	PAWR	TFPI2	CDCA3	HMGB2	PON2	CEP55	PHB	PTX3	RPLP2
36	QPRT	MT2A	COL4A1	CDH6	KIF20B	SIVA1	C5orf38	TUBB4B	PSMB6	PDLIM2	RPS2
37	SERF2	KCNK3	AKR1B1	MGST1	CENPW	SRSF7	IMPA2	SMC2	TPM3	CAVIN3	RPS3
38	TMEM30A	CPD	TSPAN1	APLP2	RAN	RRM1	LINC01116	DLGAP5	HSP90B1	COX7A1	RPL3
39	S100A10	PTPN1	FXYD6	VAMP5	DEPDC1	CHAF1A	AC004982.2	CCNB1	NME1	HHIP	RPLP0
40	IL18	FGFR1	ATP1A1	TSC22D1	HSP90AA1	CENPM	IFITM2	HNRNPD	EFHD2	CREB3L1	RPL4
41	JAK1	LPP	RND3	KCNQ1OT1	PLK1	MCM6	GLRX	CDC20	KRT8	CDC42EP3	RPS20
42	ATOX1	DBI	CDA	CYP3A5	HSPD1	PTMA	LAMC2	DNMT1	HSP90AB1	EVA1B	RPS12
43	SERPINE1	AC003092.1	SCNN1A	RARRES3	KIF4A	RANBP1	FXYD2	H2AFV	STOML2	CALD1	RPS6
44	TMSB10	GLDC	RBP1	TPT1	TACC3	SRSF2	COL7A1	CENPM	RPS26	CDH13	RPSA
45	CCND1	SLCO4C1	H3F3A	CA12	RAD21	HMGN2	CRNDE	UBE2S	RAN	SRM	RPL18A
46	DYSF	AHNAK2	FOLR1	FTH1	SFPQ	TUBA1B	IVNS1ABP	HMMR	ANXA2	ALDH1L2	HINT1
47	SCD	MT-CO2	SSC4D	TNFRSF1A	GTSE1	HMGB1	UCHL1	ANP32B	SERBP1	MYDGF	RPS25
48	SLN	MT-ND4	LOXL1	TGM2	STMN1	BARD1	SCNN1A	CCNA2	SNRPE	MEIS2	RPS5
49	RPL34	OGFRL1	MAL	TYMP	ARL6IP1	HNRNPD	FKBP1A	CBX5	LDHA	ITGA5	RPL31
50	COMMD6	FTL	HOXB-AS3	EEF2	DEK	PA2G4	PLEKHA1	RRM1	HNRNPA3	DKK1	RPL24

Supplementary Table 1. List of the first 50 marker genes of human proximal tubular cell (hPTC) clusters. Light orange boxes represent genes shared with cluster 8 from mouse proximal tubular cells (PTC).

	0	1	2	3	4	5	6	7	8	9
1	Slc5a2	Atp11a	Gatm	Cyp4b1	Cox4i1	Nudt19	Cyb5a	Mylk	Ptma	Tmsb4x
2	Ccdc141	Slc6a18	Car2	Gm45792	Spink1	Cndp2	Miox	Zbtb20	H2afz	H2-D1
3	Slc6a19	Slc7a13	Aldob	Slc22a8	Cox8a	Gclm	Ubb	Luc7l2	Stmn1	Cd52
4	Slc5a12	Slco1a1	Mif	Timp3	Cox6a1	Prdx1	Ttc36	Slc22a30	Ran	Srgn
5	Snhg11	Slc27a2	Ftl1	Ces1f	Cox7c	Cyb5a	Rida	Pkhd1	Ube2s	Btg1
6	Maf	Ly6a	Slc25a4	Slc22a28	Cox7a2	Ttc36	Cycs	Ddx17	Tuba1b	Ptprc
7	Fut9	Slc5a8	Oat	Car12	Guca2b	Rida	Fxyd2	Slc22a28	Cdk1	H2-K1
8	Slc34a3	Napsa	Atp5g1	BC005561	Fth1	Lap3	Scp2	Tef	H2afv	Coro1a
9	Chka	Kap	Atp6v1g1	Malat1	Uqcr11	Mpv17l	Akr1a1	Trim7	1810037I17Rik	Junb
10	Slc4a4	Ggt1	Fxyd2	Slc47a1	Ndufa4	Gstm1	Akr1c21	Gm42418	Birc5	B2m
11	Folh1	Slc22a6	Ass1	Atp11a	Dbi	Acy3	Prdx1	Vegfa	Tubb5	Fxyd5
12	Slc7a8	Mep1a	Tpt1	Slc17a3	Gpx1	Gpx4	Ass1	Ogt	Hmgb1	Shisa5
13	Cgnl1	Lpl	Akr1a1	Mlxipl	Cox6c	Cda	Eef1a1	Pnisr	Gclm	Lcp1
14	Kcnq1ot1	Slc17a3	Ldhb	Slc34a1	Cox6b1	Dbi	Eif1	Fus	Ywhah	Laptm5
15	Gm26917	Mat2a	Park7	Gm26917	Ndufb9	Mpc1	Pecr	Rsrp1	Hmgb2	Sh3bgrl3
16	Spp2	Cd36	Oxct1	Slc22a30	Cox7b	Acadm	Gapdh	Itpr1	Dut	Rac2
17	Plekha1	Slc22a30	Rpl37a	Tns1	Tpt1	Hibadh	Slc25a5	Nisch	Dek	Arhgdib
18	Car12	Cyp4b1	Gpx1	Zbtb20	Ass1	Acaa1b	Dbi	Rbm25	Ccdc34	Ier2
19	Timp3	Nat8f6	Rps29	Snx29	mt-Cytb	Eci3	Aldob	Phldb2	H3f3a	H2-Q7
20	Malat1	Ces1f	Atp5e	Cyp4a12a	mt-Nd4	Me1	Msrb1	Gm45792	Hmgn1	Cd53
21	Adra1a	Nat8	Rpl28	Neat1	Rplp1	Suclg1	Tpt1	Srsf11	Ube2c	Psmb8
22	Neu1	Cyp2e1	Cox5b	Col4a4	Ndufa13	Msrb1	Acy3	Dst	Rrm2	AW112010
23	Steap2	Ces2c	Fbp1	AC160336.1	mt-Atp6	Txn1	Prdx5	Mlxipl	S100a11	Lsp1
24	Slc7a7	Ly6e	Slc25a3	Pde4d	Prdx5	Slc25a39	Mpc1	Kcnq1ot1	Spc25	Ifngr1
25	Snx29	Agps	Acaa2	Cyp2j13	mt-Co3	Scp2	Cystm1	Tet2	Nudc	Adgre5
26	Slc43a2	Slc22a2	Idh1	Kcnj15	Cox5b	Bphl	Ybx1	Ash1l	Pbk	Ltb
27	Nktr	Mlxipl	Atp5h	Enpp2	mt-Nd2	Prdx5	Chchd2	Slc6a6	Calm2	Zfp36l2
28	Cpeb3	Gm45792	Pcbd1	Syne2	Cox5a	Ech1	Gpx1	Zcchc7	Hmgn2	Btg2
29	Ptprd	Slco3a1	Chchd2	Arhgap5	Ttc36	Acat1	Suclg1	Pde4d	Pclaf	Actb
30	Slc3a2	Slc22a12	Uqcrh	Slc5a2	Atp5j2	Vdac1	Park7	Slc25a36	Clic1	Cytip
31	Kif12	Slc17a1	Rpl36	Trabd2b	Uqcrh	Keg1	Hist1h2bc	Nmrk1	Cdca3	Ly6e
32	Cpeb4	Acsm3	Atp5a1	Cyp2e1	Cyba	Phyh	Txn1	Nuak2	Nucks1	Tmsb10
33	Ogt	Ddx5	Cox6c	Igf1r	Rpl39	Them7	Vdac1	Slc6a20b	Rps27l	Stk17b
34	Neat1	Ugt3a2	Serf2	Slc22a6	Hint1	Etfb	Ech1	Abcc2	Tubb4b	Hcst
35	Ank3	Zbtb20	Rps8	Pnisr	Atp5l	Acadl	Txndc17	Cspp1	Ranbp1	Itgal
36	4921539H07Rik	Col4a3	Rps24	Slc13a3	mt-Nd1	Cystm1	Gnas	Clk1	Tk1	Selplg
37	Atp2a2	Slc47a1	Fabp3	Atp2a2	Rps29	Eif5a	Cda	Ttc14	Cdc20	S100a6
38	Cyp2d26	Tmigd1	Mdh1	Golga4	Atpif1	Pecr	Cisd1	Cpeb4	Ccna2	Dusp2
39	Prodh2	Neat1	Atp5o-1	Slc4a4	Ndufa6	Gapdh	Akr7a5	Prpf38b	Ppia	Dock2
40	Cttnbp2	Tmem176a	Atp5g3	Col4a3	Fxyd2	Adh1	Uqcrfs1	Tmem245	Dbi	Ptpn18
41	Srrm2	Slc5a10	Rplp0	Prpf4b	Eef1a1	Rps2	Hibadh	Dgkh	Tyms	Arpc1b
42	Col27a1	Stx16	Uqcrb	Ggt1	Ndufa2	Khk	Atp5b	Tfcp2	Txn1	Ms4a4b
43	Golga4	Ghr	Rpl23	AC149090.1	Gpx4	Amacr	Mdh1	Krit1	Nusap1	Ptprcap
44	Rab11fip3	Trim7	Atp5l	Slc5a12	Rpl41	Uqcrfs1	Keg1	Trps1	Snrpe	B4galnt1
45	AC149090.1	Nudt19	Rpl19	G6pc	mt-Co2	Pank1	Chchd10	Kidins220	Ubald2	Ier5
46	D630023F18Rik	Serpinf2	Rps23	Ptprk	Rps21	Akr1c21	Ldhb	Snx29	Actg1	Itga4
47	Syne2	Tmem205	Atp5k	Slc6a6	Rps19	H2afj	Gabarap	Malat1	S100a10	Gimap6
48	Itpr2	Abcc2	Chchd10	Slc6a19	Uqcrq	Iscu	Tpi1	Lrp2	Cks2	Apbb1ip
49	Rsrp1	Slc13a3	Rps21	Trpm7	Ndufb8	Rpl17	Rpl6	Gm26917	H2afx	Rps27
50	Luc7l2	Tmbim6	Uqcr10	Vegfa	2010107E04Rik	Gnas	Dhrs4	Rbm39	Hmmr	Celf2

Supplementary Table 2. List of the first 50 marker genes of mouse proximal tubular cell (PTC) clusters. Light orange boxes represent genes shared with polyploid clusters of human proximal tubular cells (hPTC).

Patients	
Number	**45**
Gender (male)	**33/45 (73.3%)**
Ethnicity (white)	**45/45 (100.0%)**
eGFR at baseline (mL/min/1.73mq)	**75.2 ±29.8**
eGFR < 90 mL/min/1.73mq	**27/45 (60.0%)**
CKD (eGFR < 60 mL/min/1.73mq) at baseline	**15/45 (33.3%)**
CKD (eGFR < 60 mL/min/1.73mq) at baseline in late biopsies stained for fibronectin	**6/16 (37.5%)**
Age at biopsy (years)	**52 ±17**
Histology	
Primary glomerular	**15/45 (33.3%)**
Secondary FSGS	**12/45 (26.7%)**
Tubular	**8/45 (17.8%)**
Vascular	**5/45 (11.1%)**
Other (DN, Transplant)	**5/45 (11.1%)**
Kidney function at biopsy	
Serum creatinine (mg/dL)	**1.8 [1.13-2.65]**
eGFR (mL/min/1.73mq)	**41.0 [25.0-70.0]**
eGFR < 60 mL/min/1.73mq	**30/45 (66.7%)**
eGFR < 30 mL/min/1.73mq	**18/45 (40.0%)**
ESKD	**0/45 (0%)**
Previous AKI episode	
Time AKI-to-biopsy (years)	**0.15 [0.03-0.49]**
Time AKI-to-biopsy (days)	**53 [10-179]**
Early group (2 – 15 days)	**14/45 (31.1%)**
Late group (>15 days)	**31/45 (68.8%)**
AKI stages<br>- AKIN 1<br>- AKIN 2<br>- AKIN 3	 **25/45 (55.6%)**<br>**11/45 (24.4%)**<br>**9/45 (20.0%)**
Two or more AKI episodes prior to biopsy	**9/45 (20.0%)**
Last follow-up (LFU)	
LFU, time from biopsy (years)	**1.3 [0.4-2.8]**
Serum creatinine LFU (mg/dL)	**1.22 [0.99–1.90]**
eGFR at LFU (mL/min/1.73mq)	**51.5 [27.4-70.1]**
eGFR < 90 mL/min/1.73mq	**40/45 (88.9%)**
CKD (eGFR < 60 mL/min/1.73mq) at LFU	**25/45 (55.6%)**
ESKD at LFU	**7/45 (15.6%)**

Supplementary Table 3. Clinical information of patients affected by CKD after AKI.
