# Supplementary material for: Tubular cell polyploidy protects from lethal acute kidney injury but promotes consequent chronic kidney disease
Source: Nat Commun. 2022 Oct 4;13:5805. doi: 10.1038/s41467-022-33110-5 (PMC9532438; doi:10.1038/s41467-022-33110-5)
Supplement: Supplementary file 3 — Reporting Summary [file 41467_2022_33110_MOESM3_ESM.pdf]

nature portfolio

Corresponding author(s): Paola Romagnani, Elena Lazzeri

Last updated by author(s): Sep 12, 2022

Reporting Summary

Nature Portfolio wishes to improve the reproducibility of the work that we publish. This form provides structure for consistency and transparency in reporting. For further information on Nature Portfolio policies, see our Editorial Policies and the Editorial Policy Checklist.

Statistics

For all statistical analyses, confirm that the following items are present in the figure legend, table legend, main text, or Methods section.

n/a	Confirmed	
☐	☒	The exact sample size (*n*) for each experimental group/condition, given as a discrete number and unit of measurement
☐	☒	A statement on whether measurements were taken from distinct samples or whether the same sample was measured repeatedly
☐	☒	The statistical test(s) used AND whether they are one- or two-sided<br>*Only common tests should be described solely by name; describe more complex techniques in the Methods section.*
☒	☐	A description of all covariates tested
☒	☐	A description of any assumptions or corrections, such as tests of normality and adjustment for multiple comparisons
☐	☒	A full description of the statistical parameters including central tendency (e.g. means) or other basic estimates (e.g. regression coefficient) AND variation (e.g. standard deviation) or associated estimates of uncertainty (e.g. confidence intervals)
☐	☒	For null hypothesis testing, the test statistic (e.g. *F*, *t*, *r*) with confidence intervals, effect sizes, degrees of freedom and *P* value noted<br>*Give P values as exact values whenever suitable.*
☒	☐	For Bayesian analysis, information on the choice of priors and Markov chain Monte Carlo settings
☒	☐	For hierarchical and complex designs, identification of the appropriate level for tests and full reporting of outcomes
☐	☒	Estimates of effect sizes (e.g. Cohen's *d*, Pearson's *r*), indicating how they were calculated

Our web collection on statistics for biologists contains articles on many of the points above.

Software and code

Policy information about availability of computer code

Data collection	Immunofluorescence data were collected with Leica SP8 STED 3X confocal microscope (Leica Microsystems). For Fibronectin, CDK4/p-H3 and ploidy quantification, whole sections were acquired using the sequential scanning on the same microscope. FACS data were collected using MacsQuant instrument (Miltenyi Biotec) or FACSAria III BD (Bioscience) for FACS sorting. Real-time analysis data were collected using LightCycler® 480 (Roche Diagnostics) or 7900HT Fast Real-Time (Applied Biosystem) for all the other Real-time data. GFR measurements were recorded using a miniaturized imager device built from two light-emitting diodes, a photodiode and a battery (Mannheim Pharma and Diagnostics GmbH, Mannheim, Germany) mounted via a double-sided adhesive tape onto the shaved animals' neck. Electron microscopy data were collected using a LKB-Nova ultramicrotome (LKB, Bromma, Sweden, www.lkb.com). Single cell RNA-seq were prepared on 10x Chromium Single Cell instrument (10x Genomics) and sequenced on an Illumina NextSeq550 (Illumina Inc., RRID:SCR_020138).
Data analysis	GFR measurements were analyzed using MPD Studio software ver.RC6 (MediBeacon GmbH Cubex41, Mannheim, Germany). FACS data were analyzed using FlowLogic 7.2.1, Inivai Technology. Fibronectin deposition, cell surface area and ploidy quantification were quantified using Image J software (RRID:SCR_003070). FACS sorting data were analysed by FacsDiva software (Beckman Coulter). For single cell RNA-seq (scRNA-seq) analysis, Cell Ranger pipeline (version 3.0.1) was used for raw data processing, Scanpy (v1.72.) and scran computeSumFactors method for data preprocessing and analysis, Monocle v. 2.14.0 to generate trajectories. Real-time data were analyzed using LightCycler® 480 (Roche Diagnostics) or 7900HT Fast Real-Time (Applied Biosystem) built in programs. Statistical analysis was performed using SPSS (RRID:SCR_002865) for Kaplan-Meier survival analyises and OriginPro (RRID:SCR_015636) statistical software for all the other analyses.

For manuscripts utilizing custom algorithms or software that are central to the research but not yet described in published literature, software must be made available to editors and reviewers. We strongly encourage code deposition in a community repository (e.g. GitHub). See the Nature Portfolio guidelines for submitting code & software for further information.

Data

Policy information about availability of data

All manuscripts must include a data availability statement. This statement should provide the following information, where applicable:

- Accession codes, unique identifiers, or web links for publicly available datasets
- A description of any restrictions on data availability
- For clinical datasets or third party data, please ensure that the statement adheres to our policy

The authors declare that all data supporting the findings of this study are available within the article and its Supplementary Information Files. Source data are provided with this paper. Processed data for all human and mouse scRNA-seq libraries generated in this study have been deposited in the Gene Expression Omnibus (GEO) database under accession code GSE212275 for the human dataset (https://www.ncbi.nlm.nih.gov/geo/query/acc.cgi?acc=GSE212275) and GSE212273 for the mouse dataset (https://www.ncbi.nlm.nih.gov/geo/query/acc.cgi?acc=GSE212273).

Field-specific reporting

Please select the one below that is the best fit for your research. If you are not sure, read the appropriate sections before making your selection.

☒ Life sciences ☐ Behavioural & social sciences ☐ Ecological, evolutionary & environmental sciences

For a reference copy of the document with all sections, see nature.com/documents/nr-reporting-summary-flat.pdf

Life sciences study design

All studies must disclose on these points even when the disclosure is negative.

Sample size	Sample sizes were based on previously conducted and published experiments in which statistically significant differences were observed between groups (Lazzeri E. et al Nature Comms 2018 and Peired AJ. et al. Science Trans Med 2020) and power analyses. Sample size of 4-10 mice was employed, unless noted otherwise. At least three biological repeats were performed for all the experiments. The number of patients included in the study was determined by the availability of patients' specimens.
Data exclusions	Fibronectin staining was carried out in biopsies belonging to the "late" group. Where kidney cortical tissue was not available, the sample was excluded from the analysis.
Replication	All attempts of replication were successful. At least three biological repeats were performed for all the experiments. Experiments were performed from different technicians indipendently. The main experimental findings were cross-validated using multiple and different methodologies (single-cell RNA-sequencing, transgenic mice, FACS, immunofluorescence, ChIP, immunohistochemistry).
Randomization	For all the experiments performed, no specific method of randomization was used. For the in vivo studies, animals with identical genotype and similar age, were assigned to experimental groups in a blinded manner.
Blinding	Bi-coloured tubular cells of the various segments in Pax8/Confetti mice, YAP1+ and TAZ+ tubular cell nuclei, beta-galactosidase+ tubular cells, CDK4+p-H3+ tubular cells, and tubular score in mice and in human biopsies were counted by two independent blinded observers. For the in vivo experiments and all the other experiments not listed above, blinding was not conducted during experiments because collected data were quantitative and not influenced by investigator's bias.

Reporting for specific materials, systems and methods

We require information from authors about some types of materials, experimental systems and methods used in many studies. Here, indicate whether each material, system or method listed is relevant to your study. If you are not sure if a list item applies to your research, read the appropriate section before selecting a response.

Materials & experimental systems

n/a	Involved in the study	
☐	☒	Antibodies
☐	☒	Eukaryotic cell lines
☒	☐	Palaeontology and archaeology
☐	☒	Animals and other organisms
☐	☒	Human research participants
☒	☐	Clinical data
☒	☐	Dual use research of concern

Methods

n/a	Involved in the study	
☒	☐	ChIP-seq
☐	☒	Flow cytometry
☒	☐	MRI-based neuroimaging

Antibodies

Antibodies used

Immunofluorescence:
anti-Fibronectin (1:100, Abcam, ab2413)
anti-Ki67 (1:50, Abcam, ab15580)
anti-aquaporin-1 (AQP1, 1:100, Millipore, AB2219)
anti-Tamm-Horsfall (THP, 1:20, Cederlane, CL1032A)
anti-aquaporin-2 (AQP2, 1:500, Abcam, Ab62628)
anti-GFP (1:100, Thermo Fisher Scientific, A-11122)
anti-phosphorylated Histone 3 (p-H3, 1:2000, Abcam, ab14955)
anti-cyclin dependent kinase 4 (CDK4, 1:50, Santa Cruz Biotechnology, SC-601)
anti-TAZ (1:100, Abcam, ab110239)
anti-active YAP1 (1:500, Abcam, ab205270)
FACS:
anti-DsRed (1:25 Clontech, 632496)
normal rabbit IgG (1:250, Thermo Fisher Scientific, 02-6102)
Alexa Fluor 647, 488 goat anti-rabbit (1:100, Thermo Fisher Scientific A-21245, A-11008)
anti-GFP-488 (1:100 Termo Fisher Scientific, A21311)
IHC:
anti-active YAP1 (1:500, Abcam, ab205270)
anti-TAZ (1:100, Abcam, ab110239)
ChIP:
anti-YAP1 (10µg/IP, Novus Biologicals, NB110-58358)
normal rabbit IgG (7µg/IP Thermo Fisher Scientific, 02-6102)

Validation

All commercially available antibodies are validated by suppliers as follows:
anti-Fibronectin: https://www.abcam.com/fibronectin-antibody-ab2413.html?productWallTab=ShowAll
anti-Ki67 https://www.abcam.com/ki67-antibody-ab15580.html KO validated, and previously validated by our group (Lazzeri E. el al. Nature Communications 2018)
anti-aquaporin-1 https://www.citeab.com/antibodies/224066-ab2219-anti-aquaporin-1-antibody and previously validated by our group (Lazzeri E. el al. Nature Communications 2018)
anti-Tamm-Horsfall https://www.szabo-scandic.com/en/anti-human-tamm-horsfall-ascites-clone-10-32-mouse-igg2b and previously validated by our group (Lazzeri E. el al. Nature Communications 2018)
anti-aquaporin-2 https://www.abcam.com/aquaporin-2-antibody-ab62628.html
anti-GFP https://www.thermofisher.com/antibody/product/GFP-Antibody-Polyclonal/A-11122 and previously validated by our group (Lazzeri E. el al. Nature Communications 2018)
anti-phosphorylated Histone 3 https://www.abcam.com/histone-h3-phospho-s10-antibody-mabcam-14955-ab14955.html and previously validated by our group (Lazzeri E. el al. Nature Communications 2018)
anti-cyclin dependent kinase 4 is no longer available but previously validated by our group (Lazzeri E. el al. Nature Communications 2018)
anti-TAZ https://www.abcam.com/taz-antibody-ab110239.html
anti-active YAP1 https://www.abcam.com/active-yap1-antibody-epr19812-ab205270.html KO validated
anti-DsRed https://www.takarabio.com/products/antibodies-and-elisa/fluorescent-protein-antibodies/red-fluorescent-protein-antibodies and previously validated by our group (Lazzeri E. el al. Nature Communications 2018)
normal rabbit IgG https://www.thermofisher.com/antibody/product/Rabbit-IgG-Isotype-Control/02-6102 and previously validated by our group (Lazzeri E. el al. Nature Communications 2018)
Alexa Fluor 647 and 488 goat anti-rabbit https://www.thermofisher.com/antibody/product/Goat-anti-Rabbit-IgG-H-L-Highly-Cross-Adsorbed-Secondary-Antibody-Polyclonal/A-21245, https://www.thermofisher.com/antibody/product/Goat-anti-Rabbit-IgG-H-L-Cross-Adsorbed-Secondary-Antibody-Polyclonal/A-11008 and previously validated by our group (Lazzeri E. el al. Nature Communications 2018)
anti-YAP1 https://www.novusbio.com/products/yap1-antibody_nb110-58358 KO validated

Eukaryotic cell lines

Policy information about cell lines

Cell line source(s)

Primary human proximal tubular cells were purchased from ATCC, catalog number PCS-400-010.

Authentication

The cell line was authenticated through scRNA-seq analysis.

Mycoplasma contamination

A certificate of the analysis is provided for each cell lot purchased.

Commonly misidentified lines
(See ICLAC register)

Name any commonly misidentified cell lines used in the study and provide a rationale for their use.

Animals and other organisms

Policy information about studies involving animals; ARRIVE guidelines recommended for reporting animal research

Laboratory animals

The Pax8.rtTA;TetO.Cre;R26.FUCCI2aR (Pax8/FUCCI2aR also defined Pax8/WT in some experiments) mouse model was obtained by crossing Pax8.rtTA;TetO.Cre mice with mice harboring the Fluorescent Ubiquitin-based Cell cycle Indicator (FUCCI2aR) Cre-

dependent reporter (European Mouse Mutant Archive (EMMA), INFRAFRONTIER-I3, Neuherberg-München, Germany). These transgenic mice were crossed with either SAV1 knock-out (Sav1tm1.1Dupa, JAX:027933) to obtain Pax8/SAV1ko mice or with YAP1 knock-out mice (Yap1tm1.1Dupa, JAX:027929) to obtain Pax8/YAP1ko mice both from the Jackson Laboratory. To induce reporter expression, at 5 weeks of age mice were given 4mg/ml doxycycline hyclate (Merck) in drinking water supplemented with 2.5% sucrose (Merck) for 10 days, followed by a 7-day washout. At the end of the washout period, male mice underwent a unilateral ischemia reperfusion injury (IRI, ischemic injury) of 30min, and were then sacrificed at day 2, 3, 5 and 30 days post-IRI. Sham operated mice were used as controls. As IRI tolerance is profoundly increased in female mice compared with that observed in male mice, female mice underwent glycerol-induced nephrotoxic AKI (nephrotoxic-AKI, nephrotoxic injury). Mice were sacrificed at day 1, 2 and 30 days after nephrotoxic-AKI. Healthy mice were used as controls. For the treatment with YAP1 inhibitor CA3 (CIL56), Pax8/FUCCI2aR mice and non-induced (WT) littermates underwent nephrotoxic-AKI (female mice) or ischemic-AKI (male mice) at 7 weeks of age. To trigger recombination of Pax8/YAP1ko and Pax8/WT mice after ischemic (male) and nephrotoxic (female) AKI, mice were given 4mg/ml doxycycline hyclate (Merck) at 7 weeks of age in drinking water supplemented with 2.5% sucrose (Merck) at 4 days after AKI for 10 days, followed by a 7-day washout. For the senolytic treatment, Pax8/FUCCI2aR mice underwent nephrotoxic-AKI (female mice) at 7 weeks of age.
The Pax8.rtTA;TetO.Cre;R26.Confetti (Pax8/Confetti) mice were developed by crossing the Confetti strain Gt(ROSA)26Sortm1(CAG-Brainbow2.1)Cle/J with the TetO.Cre strain B6.Cg-Tg(TetO-Cre)1Jaw/J, both purchased from Jackson Laboratory (Bar Harbor, ME, USA). Double transgenic mice were then crossed with a Pax8.rtTA mouse. At 7 weeks of age heterozygous male Pax8/Confetti mice underwent IRI to trigger polyploid TC formation and 13 days after damage they were given 4mg/ml doxycycline hyclate followed by 7-day washout. All mice were developed on a full C57BL/6 background. Mice were housed in a specific pathogen-free facility with free access to chow and water and a 12-hour day/night cycle.

Wild animals	The study does not involve wild animals.
Field-collected samples	The study does not involve samples collected from the fields.
Ethics oversight	Animal experiments were approved by the Institutional Review Board and by the Italian Ministry of Health and performed in accordance with institutional, regional, and state guidelines and in adherence to the National Institutes of Health Guide for the Care and Use of Laboratory Animals. The references to the ethics approvals are the following: 809/2017-PR; 272/2018-PR; 689/2019-PR; 864/2021-PR and 239/2022-PR. See Methods.

Note that full information on the approval of the study protocol must also be provided in the manuscript.

Human research participants

Policy information about studies involving human research participants

Population characteristics	Population characteristics are detailed in Supplementary Table 3.
Recruitment	All consecutive patients with different nephropathies who underwent renal biopsy in our Nephrology Units (Nephrology, Dialysis and Transplantation, Azienda Ospedaliero-Universitaria Careggi and Nephrology and Dialysis, Meyer Children's University Hospital, Florence, Italy) between 2012 and 2021 were screened in this study. Exclusion criteria were: 1) Positive immunofluorescence staining suggestive of immune-mediated nephropathy; 2) Less than two creatinine values within 48h in the period before renal biopsy which does not allow the diagnosis of AKI; 3) Insufficient clinical information and no or lacking laboratory findings. Patients were recruited upon obtaining a signed informed consent.
Ethics oversight	Autorization and approval to this study was granted by the Ethical Committee on human experimentation of the Azienda Ospedaliero- Universitaria Careggi (Clinical Trial Center (CTC) AOU Careggi, authorizations: n. OSS_10243) and by the Meyer Children's University Hospital, Florence, Italy (Clinical Trial Office, Meyer, authorizations: n.150/2016).

Note that full information on the approval of the study protocol must also be provided in the manuscript.

Flow Cytometry

Plots

Confirm that:

- [x] The axis labels state the marker and fluorochrome used (e.g. CD4-FITC).
- [x] The axis scales are clearly visible. Include numbers along axes only for bottom left plot of group (a 'group' is an analysis of identical markers).
- [x] All plots are contour plots with outliers or pseudocolor plots.
- [x] A numerical value for number of cells or percentage (with statistics) is provided.

Methodology

Sample preparation	Kidneys were processed to obtain a single cell suspension. Incubation with anti-DsRed pAb (ClonTCh, 632496) or isotype control was followed by Alexa Fluor 647 goat anti-rabbit as secondary antibody to detect mCherry+ tubular cells, whereas for detection of mVenus+ tubular cells was used an anti-GFP-488 pAb (Termo Fisher Scientific, A21311). Cells were then incubated with DAPI to perform the DNA content analysis.<br>Human proximal tubular cells transduced with pRetroX-G1-Red (mCherry-G1) retrovirus were trypsinized at passage 2 after transduction. For regular Facs analysis the protocol was the above mentioned one. In the verteporfin experiment, Alexa Fluor

	488 goat anti-rabbit was used as secondary antibody to detect mCherry+ tubular cells. For FACS sorting, the cells were fixed with PFA 0.25%, 0.5% saponin with the addition of RNAase inhibitor. Then anti-DsRed pAb (Clontech, 632496) or isotype control was incubated for 1h at RT followed by 1h incubation with secondary antibody Alexa Fluor 647 goat anti-rabbit to detect mCherry+ human proximal tubular cells. All the antibodies were diluted in 0,5% saponin with the addition of 1:100 RNAase inhibitor. All the solutions were diluted in RNAase-free PBS prepared with DEPC water. The procedure was carried out on ice. Finally, human proximal tubular cells were incubated with DAPI to perform the DNA content analysis and sorted on the FACSAria III BD.
Instrument	MacsQuant instrument (Miltenyi Biotec). Alexa Fluor 647 secondary antibody was excited by a 633nm laser line, GFP and Alexa Fluor 488 secondary antibody was excited by a 488nm laser line, DAPI was excited by a laser at 405nm. FACSAria III BD instrument (BDBioscience). Alexa Fluor 647 secondary antibody was excited by a 633 nm laser line, DAPI was excited by a 405 nm laser line.
Software	Data were analysed by FlowLogic and FACSDiva softwares.
Cell population abundance	Following cell sorting the polyploid hPTC were between the 15% and 20% percentage of the sorted mCherry+ cells.
Gating strategy	The gating strategy for the analysis of Pax8/FUCCI2aR mice is shown in Supplementary Fig. 1. Cell doublets were excluded from the analysis as previously reported by our group (Lazzeri E. et al Nat Comms 2018) and shown in Supplementary Fig.1. The gating strategy for human proximal tubular cells transduced with pRetroX-G1-Red is shown in Supplementary Fig. 3.

☒ Tick this box to confirm that a figure exemplifying the gating strategy is provided in the Supplementary Information.
